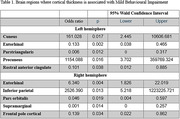# Association between Cortical Thickness and Mild Behavioral Impairment in a Southeast Asian Population ‐ Biomarkers and Cognition Study, Singapore BIOCIS

**DOI:** 10.1002/alz.092192

**Published:** 2025-01-03

**Authors:** Yi Jin Leow, Ashwati Vipin, Pricilia Tanoto, Mohammed Adnan Azam, Gurveen Kaur Sandhu, Faith Phemie Hui En Lee, Smriti Ghildiyal, Shan Yao Liew, Isabelle Yu Zhen Tan, Nagaendran Kandiah

**Affiliations:** ^1^ Lee Kong Chian School of Medicine, Nanyang Technological University, Singapore Singapore

## Abstract

**Background:**

Mild Behavioral Impairment (MBI) is the onset of sustained neuropsychiatric symptoms that are considered as a possible precursor to neurodegenerative conditions, especially dementia. The concept of MBI recognizes that behavioral changes may be an early sign of brain changes due to neurodegeneration. Very recent research has shown behavioral changes in MBI might be linked to changes in brain structure, including cortical thickness. Cortical thickness is associated with various neurodegenerative diseases, including Alzheimer’s disease and other forms of dementia. We examined the association between cortical thickness and neuropsychiatric symptoms(NPS) in a multi‐ethnic Southeast Asian population.

**Method:**

657 participants were recruited from the community living in Singapore. 399 were cognitively unimpaired (CU), 253 with mild cognitive impairment (MCI), and overall, the mean age = 59.86 years, mean education = 14.52 years with 40.2% males.

Participants completed the Mild Behavioural Impairment‐Checklist (MBI‐C), a self‐reported questionnaire. Mild Behavioural Impairment(MBI) classification was based on established clinical cut‐offs of 8.5.

Participants underwent magnetic resonance imaging (MRI) scan on a 3T Siemens Prisma Fit (Siemens, Erlangen, Germany). Structural MRI was acquired using T1‐weighted MPRAGE sequence. Structural images were preprocessed using FreeSurfer (https://surfer.nmr.mgh.harvard.edu/, software version 7.2). For each participant’s T1 images, automated segmentation and cortical parcellation was carried out using the FreeSurfer “recon‐all” processing stream, which included motion correction, removal of nonbrain tissue, automated Talairach transformation, intensity correction, volumetric segmentation, and cortical surface reconstruction and parcellation.

**Result:**

In the left hemisphere, the cortical thickness of the cuneus, entorhinal, parstriangularis, precuneus, rostral anterior cingulate, was significantly associated with the likelihood of being classified as MBI. The right – entorhinal, inferior parietal, pars orbitalis, supramarginal and frontal pole cortical thickness was significantly associated with the likelihood of being classified as MBI.

**Conclusion:**

The results highlight the importance of MBI as potential early indicators of cognitive impairment and suggest that these impairments are associated with changes in cortical thickness and increased risk of cognitive decline. The complex interplay between behavioral and cognitive symptoms in aging and neurodegenerative diseases needs to be defined.